# Potentially commercializable nerve guidance conduits for peripheral nerve injury: Past, present, and future

**DOI:** 10.1016/j.mtbio.2025.101503

**Published:** 2025-02-05

**Authors:** Chundi Liu, Mouyuan Sun, Lining Lin, Yaxian Luo, Lianjie Peng, Jingyu Zhang, Tao Qiu, Zhichao Liu, Jun Yin, Mengfei Yu

**Affiliations:** a Stomatology Hospital, School of Stomatology, Zhejiang University School of Medicine, Zhejiang Provincial Clinical Research Center for Oral Diseases, Key Laboratory of Oral Biomedical Research of Zhejiang Province, Cancer Center of Zhejiang University, Engineering Research Center of Oral Biomaterials and Devices of Zhejiang Province, Hangzhou, 310000, China; b The State Key Laboratory of Fluid Power and Mechatronic Systems, Key Laboratory of 3D Printing Process and Equipment of Zhejiang Province, School of Mechanical Engineering, Zhejiang University, Hangzhou, 310027, China

**Keywords:** Nerve guidance conduit, Peripheral nerve, Clinical efficacy, Biomaterial, Add-on strategy

## Abstract

Peripheral nerve injuries are a prevalent global issue that has garnered great concern. Although autografts remain the preferred clinical approach to repair, their efficacy is hampered by factors like donor scarcity. The emergence of nerve guidance conduits as novel tissue engineering tools offers a promising alternative strategy. This review aims to interpret nerve guidance conduits and their commercialization from both clinical and laboratory perspectives. To enhance comprehension of clinical situations, this article provides a comprehensive analysis of the clinical efficacy of nerve conduits approved by the United States Food and Drug Administration. It proposes that the initial six months post-transplantation is a critical window period for evaluating their efficacy. Additionally, this study conducts a systematic discussion on the research progress of laboratory conduits, focusing on biomaterials and add-on strategies as pivotal factors for nerve regeneration, as supported by the literature analysis. The clinical conduit materials and prospective optimal materials are thoroughly discussed. The add-on strategies, together with their distinct obstacles and potentials are deeply analyzed. Based on the above evaluations, the development path and manufacturing strategy for the commercialization of nerve guidance conduits are envisioned. The critical conclusion promoting commercialization is summarized as follows: 1) The optimization of biomaterials is the fundamental means; 2) The phased application of additional strategies is the emphasized direction; 3) The additive manufacturing techniques are the necessary tools. As a result, the findings of this research provide academic and clinical practitioners with valuable insights that may facilitate future commercialization endeavors of nerve guidance conduits.

## Graphical abstract

In this review, the commercialization process and potential development directions of nerve guidance conduits are thoroughly discussed from the laboratory stage to the clinical application stage. First, the efficacy of NGCs approved by the U.S. Food and Drug Administration is comprehensively analyzed, and a critical window period for evaluating the efficacy of NGCs is initially proposed. In addition, an in-depth analysis of the directions for conduits' clinical translation is presented, with biomaterials and additional strategies as key factors in nerve regeneration. Finally, a general outlook for future commercialization is provided through a discussion of the development path and manufacturing techniques. Combining perspectives of the past, present, and future, it is clear that the core development goals and the key core technologies can promote the effective integration of the upstream and downstream of the industrial chain. In this way, a stable and optimized commercial industry circle can be formed. In all, this review provides valuable forward-looking insights to guide the future commercialization of NGCs, particularly in experimental innovation and clinical research.

**Figure:**
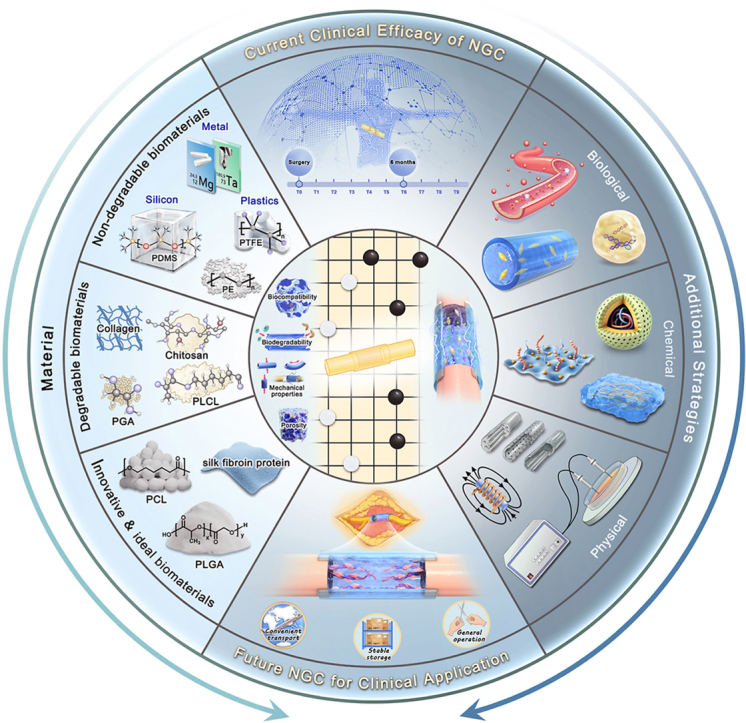

## 1 Introduction

Peripheral nerve injury (PNI) is an increasingly prevalent disorder caused by accidents, trauma, medically induced injuries, and other causes [1]. PNI has a relatively high annual incidence of 13–23 per 100,000 individuals in developed countries [2]. Although peripheral nerves have certain self-repair and regeneration abilities compared to the central nerves [3], their inherent ability to repair remains limited, thereby hindering efficient restoration [4,5]. PNI may cause a complete or partial loss of function, or even disability in the area innervated by the damaged nerve, severely affecting the patients’ daily lives [6].

Peripheral nerve discontinuities with substantial loss of nerve tissue require surgical intervention [7,8]. Microsurgical techniques including primary neurorrhaphy and nerve autograft are the standard methods [9]. Primary neurorrhaphy is preferred to restore short nerve gaps with low tension (<5 mm) in human nerve injuries [10]. Nevertheless, sutures may increase the risk of trauma, inflammation, and chronic pain [9,11]. For longer nerve gaps, worldwide nerve regeneration products are being applied. Of these, nerve autograft is currently the gold standard treatment due to its high biocompatibility, low immunogenicity, and good repair ability [12]. However, limitations, including donor shortage, donor site morbidity, donor-recipient mismatch, and neuroma, still exist. In addition to nerve autograft, there are alternative nerve repair methods. Nerve grafts including allografts (e.g. Avance Nerve Graft® approved in the United States [13], Acellular allogeneic nerve repair material® approved in China [14]) and xenografts enlarge the donor source but carry the risk of harmful immune responses [15,16]. Neural tissue engineering is an innovative and viable therapeutic alternative and is increasingly used for peripheral nerve regeneration. It primarily refers to repairing nerve defects by constructing nerve guidance conduits (NGCs).

NGCs have undergone an ongoing evolution. The NGCs developed since the early stages mainly refer to simple hollow conduits that bridge nerve gaps and support axonal extensions. NGCs mainly in simple hollow form have been brought into clinical use. Till now, the United States Food and Drug Administration (FDA) has approved 34 nerve regeneration devices (product code: JXI) [17], and several of these are NGCs repairing nerve transection injuries with substantial tissue loss. The FDA-approved NGCs include natural and synthetic polymer conduits: (1) collagen-based (K011168, K012814, K130557, and K163457); (2) chitosan-based (K143711 and K180222); (3) poly(glycolic acid) (PGA)-based (K983007); (4) poly(d,l-lactide-co-e-caprolactone) (PLCL)-based (K032115, K050573, K112267, and K230794). China's National Medical Products Administration (NMPA) has also approved several NGCs based on collagen (20163132399), chitosan, gelatin, and PLGA (20203130898) [14].

In the ongoing development of neural tissue engineering, it is insufficient to repair nerve defects with a single scaffold. The classic three elements of tissue engineering refer to scaffold materials, seed cells, and bioactive factors [18]. Based on this classical theory, the five elements of neural tissue engineering have been creatively proposed as biomaterials, cells, factors, extracellular matrix, and regenerative microenvironment [[19], [20], [21], [22]]. The connotations of the NGCs have been enriched into polymer-based or ECM-based biomaterial conduits that combine additional strategies to repair nerve damage. Tissues and implants cooperate to form a regenerative microenvironment with a dynamic balance of biochemical and biomechanical cues, leading to better nerve repair. Studies on multifunctional NGCs have been conducted extensively in laboratory animals including rats [23], rabbits [24], dogs [25], and pigs [26]. Biological cues (seed cells [6,27] chemical cues (factors [28,29]), and physical cues (topological structures and physical fields [[30], [31], [32], [33]]) can regulate regenerative cells, events, and microenvironment through different mechanisms. However, several practical factors affect the clinical translation of these laboratory products due to the more complex and demanding application environments in humans. Biological strategies may carry risks such as limited cell acquisition, cell leakage, and immune rejection [34,35]. Chemical agents have short half-lives and can be easily inactivated, hindering the storage, transportation, and application of the conduits [36,37] External physical signals face challenges in achieving non-invasive and targeted therapies [38]. Morphological designs have higher realizability and require advanced manufacturing techniques for custom and steady production [39,40] Therefore, it is necessary to select and optimize suitable multifunctional products before their application to humans.

As the most potential alternative product, the commercialization of nerve guidance conduit (NGC) is a critical goal in the field of nerve regeneration. This process continues throughout the product lifecycle from laboratory to clinical [13]. Academic and clinical professionals seeking commercialization may encounter obstacles in comprehending the connection between clinical and laboratory phases, as well as determining the positioning and potential of various products. To address these inquiries, we aim to assess the current performance of clinical products, direct further trials, track laboratory research progress, and identify potential products for future commercialization development. In this work, we employ a comprehensive data analysis to evaluate the efficacy of FDA-approved NGCs, which also provide target recommendations to enhance future research. We discuss the research progress of laboratory NGCs, with an emphasis on biomaterials and add-on strategies, as well as their limitations and possibilities for clinical translation. On this basis, the development path for commercialization of NGCs is discussed prospectively to serve as a reference for academic and clinical workers concerned with commercialization.

## 2 Comprehensive analysis of NGC clinical efficacy

**Fig. 1:**
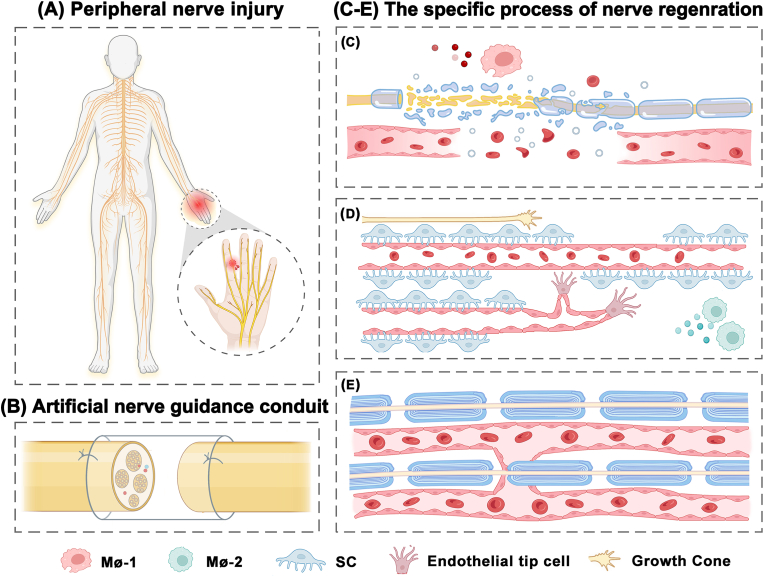
Diagram peripheral nerve injury repair (A) with artificial nerve guide conduit (B). (C–E) Nerve regeneration processes after injury. (C) After the injury, Waller degeneration occurs, axons are disintegrated, myelin is shed, and Schwann cells (SCs) transform into a repair phenotype, recruiting macrophages M1 (Mø-1) to enter the site of injury, followed by joint phagocytosis and removal of axon and myelin debris. (D) Macrophages are polarized into M2 type (Mø-2) in response to a hypoxic environment, which stimulates endothelial cell (EC) proliferation, migration, and budding to form new blood vessels. The new vasculature system acts as a physical track to guide SC migration. The reprogrammed SCs extend lengthwise to form Büngner bands. The axon tip forms a new growth cone and is directed to regenerate under the guidance of SC. (E) The microvascular network in the nerve is reconstructed, SCs are redifferentiated to form myelin, and the regenerated axons are remyelinated, representing that the repair was completed [38].

A meta-analysis of the efficacy of FDA-approved NGC was performed. The digital nerve was used as a model of PNI owing to the high incidence of digital nerve injury [10], high homogeneity of digital nerve function measurements, and widespread use of NGCs in digital nerve repair. The concrete repair events occurring at the site of a digital nerve injury after treatment with an NGC are shown in Fig. 1 [41].

**Fig. 2:**
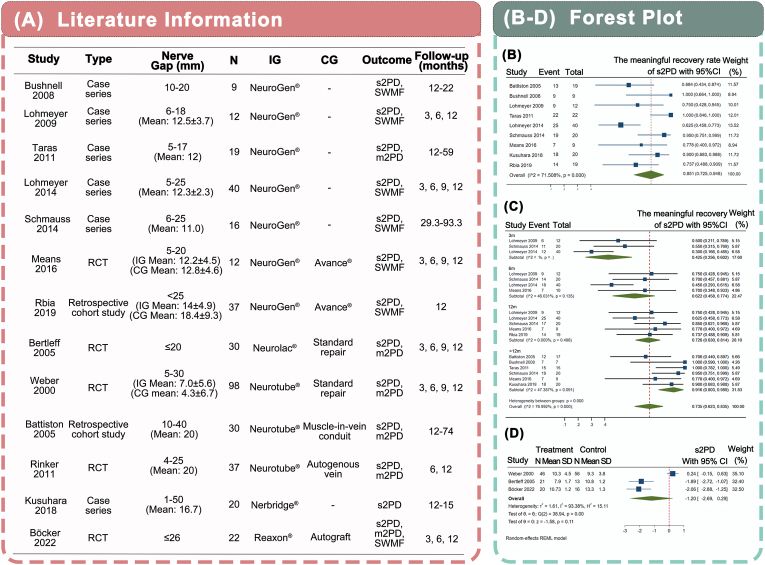
(A) Basic information on the 13 included studies. (B–D) Forest plot. (B) Effect of FDA-approved NGCs on s2PD. (C) Subgroup analysis of the effect of FDA-approved conduits on the recovery rate of s2PD based on the follow-up duration. There were four subgroups: 3 months (3m); 6 months (6m); 12 months (12m); and longer than 12 months (>12m) according to the duration of follow-up. (D) Comparative analysis of the efficacy of FDA-approved artificial conduits versus standard repairs on s2PD. N, number of participants; IG, intervention group; CG, control group.

In June 2024, 561 records were searched from the databases. After excluding duplicate studies and those that were beyond the inclusion criteria, 13 articles remained [[42], [43], [44], [45], [46], [47], [48], [49], [50], [51], [52], [53], [54]] (Fig. S1A). The included trials encompass four main types of NGCs: collagen-based (Neurogen/Neuragen), chitosan-based (Reaxon Plus), PGA-based (Neuralac, Nerbridge), and PLCL-based (Neurotube). The length of the nerve gaps varied, and most of the defects did not exceed 25 mm. Sensory functions, including static two-point discrimination (s2PD), moving two-point discrimination (m2PD), and Semmes-Weinstein Monofilament testing (SWMF), were used as the outcome evaluation indexes. Most of the follow-up periods were 3, 6, 9, and 12 months (Fig. 2A).

A meta-analysis based on single-arm evidence was performed to statistically analyze the clinical role of NGCs using the method described in the Supporting Information. The statistical results demonstrated a good recovery of sensory function after NGC repair (Figure S1C, Fig. 2B, Fig. S1D, Fig. S1E).In the rate analysis, s2PD showed a high meaningful recovery rate of 85.1 % but exhibited large heterogeneity (heterogeneity I2 = 71.508 %, Fig. 2B). According to the subset analysis, the follow-up duration was a crucial source of heterogeneity (Fig. 2C). The result showed a 60 % functional recovery rate of s2PD at 6 months of follow-up, which increased by only 10 % (to approximately 70 %) at 12 months. Nerve function recovered rapidly within the first 6 months after conduit implantation, making the effect easily observable. Therefore, we propose that the 6-month follow-up period may be a pivotal window for evaluating the efficacy of NGC as a reference in subsequent clinical studies.

Additionally, a meta-analysis of three randomized controlled trials (RCTs) among the included studies was performed to investigate the differences between NGCs and standard repairs (Fig. 2D) [[42], [43], [44]]. The results revealed no statistical difference between the artificial NGC and standard repair. Nevertheless, the statistical heterogeneity was large owing to the insufficient sample size. Further trials should be carried out to strengthen this hypothesis. Increasing the number of RCTs, trial subjects, and outcome indicators can help evaluate the efficacy of commercial NGC products and provide guidance for clinical treatment and further research.

In summary, we evaluated the clinical efficacy of FDA-approved NGCs and compared their repair effects to those of standardized repair techniques. Combining clinical practice and scientific research can provide clues for exploring future development trends and directions for NGCs.

## 3 Research progress and key influencing factors of laboratory NGCs

Bibliometrics and data visualization lay the foundation for analyzing the current situation and prospects of NGC applications. Keywords represent condensed topics from an article set in the relevant domain [55]. Thus, core research topics of NGC applications were extracted using a keyword co-occurrence analysis. Literature was quantitatively visualized using CiteSpace (Version 6.2.4) to track research hotspots related to peripheral NGCs since the 21st century. The specific methods used are described in Supporting Information.

**Fig. 3:**
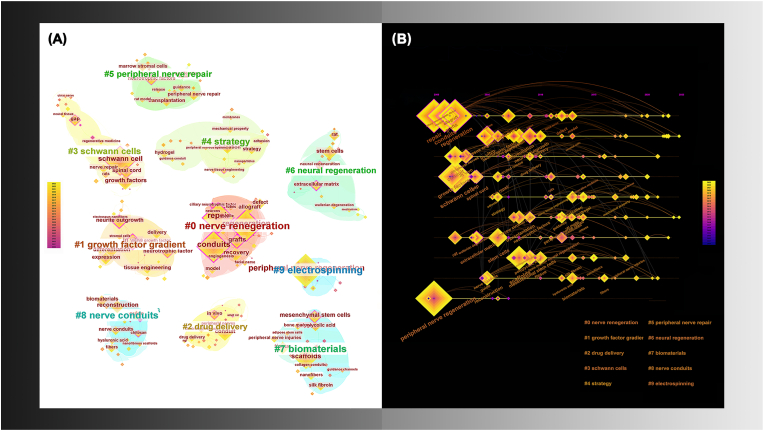
Keywords cluster (A) related to peripheral nerve guidance conduit and time diagram (B), including 10 clusters from #0 to #9. Keyword nodes can be observed under each cluster; the size of the nodes represents the keyword frequency, while the node color reflects the time development of the keyword. (For interpretation of the references to color in this figure legend, the reader is referred to the Web version of this article.)

Fig. 3A shows that all keywords were classified into 10 clusters. In addition to several clusters directly related to nerve regeneration (#0), peripheral nerve repair (#5), and neural regeneration (#6), the remaining clusters focused on conduit fabrication and related biomaterials (#7 biomaterials, #8 nerve conduits, and #9 electrospinning), as well as modification methods for conduits (#1 growth factor gradient, #2 drug delivery, and #4 strategy). Materials such as collagen, chitosan, and polyglycolic acid (PGA) have been used in most studies. Clusters #1 and #2 provided crucial concepts of biological and chemical modification, whereas clusters #7, #8, and #9 considered physical cues, including fiber and channel morphology. Additionally, cluster #3 suggested that Schwann cell (SC) should be prioritized for PNI repair. Furthermore, the timing diagram indicated that “material selection for NGC fabrication” and “modification” have gradually become the hot spot of nerve regeneration research (Fig. 3B).

### 3.1 Material

Biomaterials are fundamental elements of NGC scaffolds. Material structure and properties influence the behavior and fate of cells and tissues and regulate nerve regeneration and functional recovery [19] Early clinical researchers have used bioinert materials, such as silicone, plastic, and metal, to repair peripheral nerves [56,57]. However, these nondegradable and non-absorbable biomaterials remain in the body as foreign bodies, inducing severe fibrotic reactions and scar tissue formation, resulting in adverse outcomes and impaired nerve regeneration [56,58]. Bioabsorbable and biodegradable materials are currently being applied in NGCs due to developments in materials science. The FDA approved some of these materials for clinical and commercial use, as reflected in the clinical studies included in the statistical analyses. The performances of the materials used in commercial NGCs are summarized in Table 1, followed by a more in-depth discussion about the advantages and disadvantages of these materials.

**Table 1:** Summarized efficacy and characteristics of nerve guidance conduits classified by different materials in the included literature.

Article	Material	Conclusion	Advantages and limitations
Bushnell 2008 [45]	Bovine type I collagen	The recovery of sensory function in collagen nerve tubes compares favorably with the various types of nerve repair and reconstruction reported in the existing literature at that time.	Advantages: Excellent biocompatibility, semipermeable, chemotaxis, flexibility, and less inflammation response Limitations: the possibility of inadequate mechanical strength
Lohmeyer 2009 [46]		Collagen conduits are confirmed as a promising neural reconstruction technique.	
Taras 2011 [47]		Collagen conduits reliably provide a repair method to restore nerve function in gaps shorter than 2 cm.	
Lohmeyer 2014 [48]		Collagen conduits are a promising technique for nerve reconstruction with gap lengths of 5–25 mm.	
Schmauss 2014 [49]		Long-term recovery of the digital nerve after collagen tube repair depends on the length of the nerve space, and the effect is better when <10 mm.	
Means 2016 [50]		Hollow collagen conduits improved functional recovery and prevented significant symptomatic neuroma formation.	
Rbia 2019 [52]		Both nerve guidance conduits and treated nerve allografts provided an effective means of reestablishing the 2.5 cm digital nerve gaps after 12 months.	
Böcker 2022 [43]	Chitosan	Chitosan-based nerve guidance conduit is safe and reliable. It is suitable for nerve injury within 26 mm of the bridge junction. During the early regeneration period, tactile sensation was significantly improved, and functional results were similar to autogenous nerve transplantation.	Advantages: good biocompatibility, mucosal adhesion ability, safety, and hyposensitivity Limitations: possibility of inadequate mechanical strength and rapid degradation
Weber 2000 [42]	PGA	The PGA conduit showed improved sensory function recovery compared to end-to-end repair when repairing a nerve space of ≤4 mm. PGA conduit also produced better results than nerve grafts when repairing larger nerve gaps.	Advantages: great mechanical properties and flexibility Limitations: low cell affinity, increased risk of complications (related to harmful degradation products)
Battiston 2005 [52]		PGA synthetic tube achieved good clinical results.	
Rinker 2011 [53]		The sensory recovery after digital nerve reconstruction with PGA conduits was comparable to that with autogenous venous conduits, and there were more postoperative complications.	
Kusuhara 2018 [54]		PGA nerve guidance conduits with internal and external coating are suitable for digital nerve repair in short nerve gaps.	
Bertleff 2005 [44]	PLCL	PLCL nerve guidance conduit is suitable for the repair of transect injuries to peripheral nerves.	Advantages: excellent biocompability, mechanical properties, degradation rate, and non-toxicity (related to less harmful degradation products) Limitations: low cell affinity, and lack of applicability to injuries in the joint region (related to strong rigidity)

#### 3.1.1 FDA-approved materials for current clinical use

##### 3.1.1.1 Natural polymers

Collagen is an essential and abundant natural component of the body [59,60] The material is highly praised for its excellent hydrophilicity, biocompatibility, biodegradability, flexibility, chemotaxis, and low antigenicity [[61], [62], [63], [64]]. Furthermore, collagen contains adhesion ligands [65] that affect cell adhesion and regulate cell behavior by interacting with integrin [66] and other cell transmembrane receptors [67]. Therefore, collagen is a suitable biomaterial for regenerative medicine. However, collagen requires a certain amount of time and cost for extraction, separation, and purification, and its mechanical strength is limited under natural conditions [59,68,69] To address the limitations, cross-linking modifications or copolymerization with other components has been applied to scaffold fabrication to improve the mechanical strength and structural stability of collagen scaffolds [65,70]. Collagen type I, the major extracellular matrix protein in peripheral nerves [71], facilitates axonal regeneration after PNI [72]. Therefore, collagen products have been extensively used for nerve regeneration, followed by clinical commercialization. NGC, made of bovine type I collagen (Neurogen/Neuragen, K011168, 2001), was the first collagen medical device approved by the FDA for nerve repair. This NGC is a flexible, pliable, and non-fragile collagen tube with good compatibility and affinity for tissues and is less prone to secondary inflammation [17] It can be drawn from the statistical analysis section of this review that Neuragen performed well in treating digital nerve injury. Also, Neurogen revealed a positive effect during forearm nerve repair in other studies [73,74] The company that launched Neuragen has also developed other collagen-based NGCs, including Neuragen 3D (K130557, 2014) and Neuragen 3D nerve guide matrix (K163457, 2017). The newly developed devices are modified and improved upon Neuragen with a porous inner matrix composed of collagen and glycosaminoglycans, providing a guiding substrate for the transport of axon extension [17] Overall, Neurogen-related studies have concluded that collagen is a suitable biomaterial for clinical and commercial application of NGCs. Clinical data for other collagen products are awaited for future publication to thoroughly understand the performance of collagen in human studies.

Chitosan, a derivative of chitin, is easy to obtain and produce [75] and is characterized by excellent biocompatibility, hydrophilicity, mucosal adhesion, safety, non-toxicity, and low immunogenicity [[76], [77], [78], [79]]. Chitosan and its degradation products support the adhesion and growth of various cells, including those of the nervous system [80]. However, chitosan has limitations, such as low mechanical strength [81], poor stability [82], and insufficient degradability [83], which must be solved by modifying or blending with other polymers [84] The FDA has approved the NGC (Reaxon Plus). It is transparent, flexible, easy to suture, and resistant to collapse [17]. The transparency of the conduit helps observe and locate the nerve tracts. Neubrech et al. [85] used a chitosan conduit to protect the nerve injury site based on primary nerve repair, which improved digital nerve regeneration. Böcker et al. [44] conducted a clinical trial to evaluate the efficacy of chitosan-based NGCs in bridging nerve stumps. This study demonstrated that chitosan conduits can achieve effects similar to those of autografts in the early repair stage. As only one published clinical study presented data about chitosan neural conduits, the clinical efficacy of chitosan conduits requires more tests in the future.

##### 3.1.1.2 Synthetic polymers

PGA is a highly crystalline, thermoplastic, biodegradable polymer with excellent mechanical properties [86,87] It is soluble in organic solvents and rapidly degrades by hydrolysis [86,88] The acidic degradation products of PGA lowered the pH at the implant site, increasing the risk of inflammatory and immune responses in the surrounding tissue [89]. Neurotube approved by the FDA is a braided, flexible PGA conduit with corrugated walls for strength and flexibility that prevent the conduit from collapsing under the occlusive force of the surrounding tissue [17] In the last century, Mackinnon et al. conducted clinical trials to demonstrate the potential of PGA conduits for efficacy in nerve regeneration [90]. In our statistical analysis, three clinical trials including two RCTs presented excellent overall recovery results for Neurotube [42,52,53] However, Rinker et al. indicated that PGA conduits may cause more complications than autogenous venous conduits, which may be related to the harmful effects of their acid degradation products [53]. The postoperative complications of PGA need to be further explored.

PLCL, a copolymer of DL-lactide and ε-caprolactone, is commercially available for nerve regeneration. Poly(L-lactide-ε-caprolactone) (PLLA/PCL), a copolymer of L-lactide and ε-caprolactone, was applied to produce NGC earlier than PLCL. However, it was limited by its slow degradation rate and secondary foreign body reactions. Subsequently, PLCL was developed to achieve faster and more complete degradation with fewer foreign body reactions [91] Additionally, changing the ratio of lactide to ε-caprolactone can help obtain excellent mechanical properties and proper degradation rates [92,93]. PLCL is a biocompatible, bioabsorbable, and flexible material that is less toxic and inflammatory than other synthetic materials [94,95]. However, they have a lower cell affinity than natural materials because of the lack of recognition sites for cell adhesion [96] The PLCL-based Neurolac was the first transparent NGC among FDA-approved nerve repair devices [17], allowing more convenience in operation and inspection [43]. Only one RCT compared the Neurolac synthetic conduit to standard surgical treatment (direct end-to-end suturing) in the literature included in our statistical analysis, indicating a similar restorative effect between the two [43]. Neurolac has been studied in other nerve injuries. Araújo et al. demonstrated excellent therapeutic effects and clinical application of conduits for forearm nerve injuries [97]. More clinical trials are needed to illustrate the efficacy of PLCL conduits in clinical practice.

#### 3.1.2 Ideal material properties and materials potential for commercialization

**Fig. 4:**
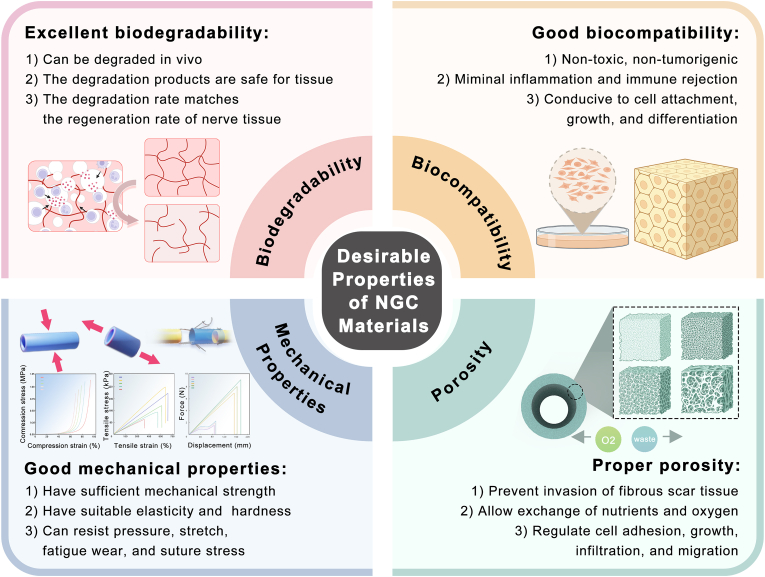
Summary of ideal NGC properties: biocompatibility; biodegradability; mechanical properties; and porosity.

The properties of a material determine its clinical applications. Consequently, clinical trials frequently indicate material performance issues to be addressed. We believe that an ideal NGC material should meet the following requirements: (1) Good biocompatibility: non-toxicity, non-tumorigenicity, non-inflammation, minimal immune rejection, and conduciveness to cell attachment and growth; (2) Excellent biodegradability: the material can be degraded in vivo, the degradation rate matches the regeneration rate of the nerve tissue, and the degradation products are safe for the tissue; (3) Good mechanical properties: the ideal material should have appropriate strength, hardness, elasticity, and flexibility to resist pressure, tension, bending, fatigue, and wear without easily breaking or rupturing and should act as a fixation and support. In addition, it should have considerable suture feasibility during surgery, exhibit minimal tearing stress during suturing, and withstand the ambulatory force during nerve regeneration; and (4) Proper porosity: this allows the diffusion and exchange of nutrients and oxygen to prevent invasion of the fibrous scar tissue [75,98,99] (Fig. 4).

Currently, NGC materials, which are expected to be commercialized, include silk fibroin, poly-ε-caprolactone (PCL), and poly (lactic-co-glycolic acid) (PLGA). These materials, which have good properties in all aspects, have been widely used in animal experiments and exhibit excellent nerve repair performances. The FDA has approved these materials as biomaterials and prepared them for commercial medical devices in other fields [[100], [101], [102]]. Silk fibroin, which is extracted from silk, is a natural and abundant polymer [103,104]. It exhibits excellent biocompatibility, adjustable biodegradability, good mechanical properties, low immunogenicity, and minimal inflammatory responses, making it a promising biological material [[105], [106], [107]]. Silk fibroin conduits have achieved nerve repair effects similar to those of autologous transplantation in animal experiments by combining topological guidance, agent delivery, and electrical stimulation [[108], [109], [110]]. PCL is a synthetic polymer extensively used in tissue engineering [111]. The biocompatible material can slowly degrade into nontoxic products after implantation. Moreover, it has better mechanical properties than other polymers, including stiffness, elasticity, and flexibility, thus playing a significant role in long-term internal support and protection in vivo [112,113]. PCL conduits, combined with different strategies, have been used in many animal experiments to repair damaged nerves, achieving recovery results comparable to those of autologous transplantation [28,112,114]. Furthermore, PLGA is an aliphatic polyester polymerized from lactic acid (LA) and glycolic acid (GA) [102]. This material is highly biocompatible and nontoxic, making it a common polyester in the biomedical field [115,116]. It can regulate the biodegradation rate by changing the LA: GA ratio and the degraded monomer products can be metabolically eliminated [116,117]. PLGA conduits have been used in injured animals and have been shown to contribute to peripheral nerve repair [[118], [119], [120]]. Additionally, all three materials mentioned above can be used for additive manufacturing [[121], [122], [123]]. Silk fibroin-based inks have been recognized as potential biomaterials for in situ three-dimensional (3D) printing and four-dimensional (4D) bioprinting via continuous improvements in manufacturing [124,125]. This may potentially lay the foundation for the future mass production of NGCs combined with additive manufacturing technology. Furthermore, the copolymers of these excellent materials can combine their respective advantages to achieve a win-win effect of the composite materials, thus effectively promoting peripheral nerve regeneration [126,127]. Hence, the innovation of materials is an entry point for the development of NGCs.

### 3.2 Additional strategies

In addition to exploring materials, various additional strategies have recently been implemented in animal experiments to construct novel multifunctional NGCs, including biological (seed-cell transplantation [6,128]), chemical (surface modification [129,130], and growth factor delivery [109,131]), and physical (3D scaffold architecture [132,133], two-dimensional (2D) surface microtopography [[134], [135], [136], [137]], and physical signal stimulation [36,138]) modifications. Based on FDA-approved materials, additional modifications to NGCs have been explored in animal experiments. The detailed discussion is as follows.

#### 3.2.1 Biological cues

Biomodification strategies based on NGCs in peripheral nerve regeneration involve seed cell transplantation [6], exogenous loading [139] or endogenous production [32] of extracellular vesicles, delivery of genes [140], and pre-vascularization [141]. Seed cell transplantation is predominant in biological methods. Schwann cells (SCs) and stem cells serve as primary seed cells for nerve regeneration. SCs reprogram to a dedifferentiated phenotype following nerve injury. This process enables the clearance of myelin and axonal debris, recruitment of macrophages, secretion of trophic factors, and formation of regenerative Büngner's bands to guide axonal extension. After axon growth, SCs differentiate into myelinating phenotypes, encasing axons to form myelinated nerve fibers, thereby restoring nerve conduction [142]. The adaptive transformation of the differentiation state of SCs is a prerequisite for nerve regeneration, while a deficiency of SCs delays and impedes the regenerative process. Researchers have sought to introduce exogenous SCs directly into nerve defects. Collagen, one of the four FDA-approved materials, is frequently utilized in studies of SC transplantation. Berrocal [143] and Burks [144] implanted autologous SCs into collagen conduits and confirmed the survival of SCs at the defect site. The inclusion of SCs enhanced nerve regeneration, leading to axon and myelin regeneration comparable to the outcomes of autologous transplantation.

**Fig. 5:**
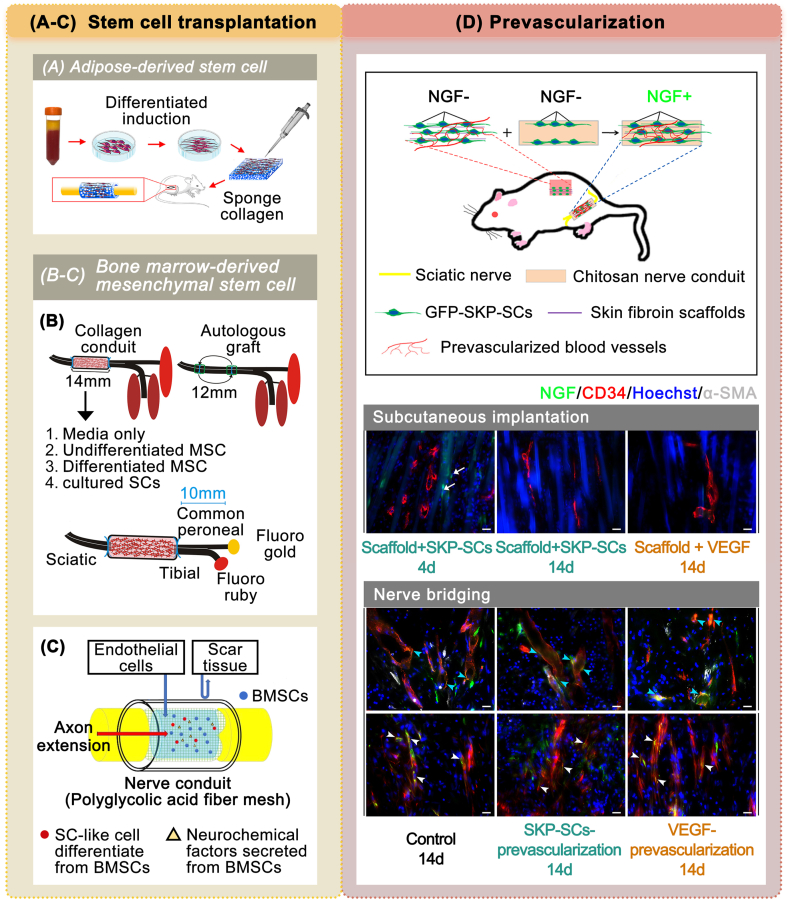
Biological modification of NGCs prepared from FDA-approved materials in animal experiments. (A–C) Stem cell transplantation for nerve repair. (A) Schematic of inductively differentiated SC-like ADSCs implanted into a collagen sponge scaffold for nerve repair (reproduced with permission) [149]. Copyright 2019, Elsevier. (B) The schematic of inductively differentiated SC-like BMMSCs implanted into a collagen scaffold for nerve repair (reproduced with permission) [147]. Copyright 2011, Elsevier. (C) Schematic of BMMSCs anchored via DABLs in a PGA conduit (reproduced under the terms of the Creative Commons Attribution 4.0 International License (CC BY 4.0)) [148]. Copyright 2023, Springer. (D) Pre-vascularization strategy for nerve repair. Schematic of subcutaneous construction of a pre-vascularized scaffold of SKP-SCs and its combined use in chitosan conduits. The expression condition of NGF after subcutaneous implantation and bridging of defects with different scaffolds is also important (reproduced with permission) [151]. Copyright 2023, Wiley.

However, the availability and proliferation capacity of exogenous SCs in vitro limits their application in nerve repair [145]. As a result, different types of stem cells have been utilized to facilitate defective nerve regeneration in two ways: orthotopic differentiation post-transplantation and transplantation following induced SC-like differentiation phenotype. Skin-derived stem cells (SDSCs) have played a role in the early stages of peripheral nerve repair. The research conducted by Marchesi et al. demonstrated that the combination of collagen conduits and SDSCs greatly improved sciatic nerve regeneration [146]. Subsequent studies have focused on bone marrow mesenchymal stem cells (BMMSCs). Ladak et al. [147] implanted BMMSCs with a previously induced SC phenotype into collagen NGCs, leading to enhanced sciatic nerve repair compared with hollow NGCs (Fig. 5B). Kakinoki et al. [148] applied a PGA fiber network conduit as an external scaffold and anchored BMMSCs with decellularized allogeneic nerve basal lamellae (DABLs) filled in the conduit cavity (Fig. 5C). These results demonstrated that the neural histomorphology index did not reach the repair level observed in autologous transplantation. Adipose-derived stem cells (ADSCs) have shown promising results in relevant research. Yu et al. [149] loaded SC-like cells derived from human ADSCs into the collagen sponge scaffold, effectively promoting nerve regeneration (Fig. 5A). Fujimaki et al. [150] injected dedifferentiated fat cells with stemness into a PGA/collagen NGC to promote defective facial nerve regeneration in rats. Various types of stem cell transplantations have been extensively utilized in studies on NGC repair. In these studies, conduits containing collagen enhanced cell adhesion and promoted cell survival, thereby leading to superior nerve regeneration compared to conduits made from other synthetic materials.

Different types of stem cells exhibit varying repair performances in vivo. Among the three stem cell types, SDSCs partially failed to survive in vivo, with only a minor proportion differentiating into myelin-supporting cells. Studies involving BMMSCs did not achieve comparable in vivo repair effects to those of autologous transplants, whereas studies involving ADSCs did. However, these findings are confined to the aforementioned studies based on clinically approved materials for NGCs. The comparative study has discovered that BMMSC and ADSC transplantation into nerve allografts showed comparable beneficial effects in nerve regeneration [152]. The actual and identifiable advantages of ADSCs include their simple and massive accessibility, high availability, and rapid proliferation [153,154].

Biological strategies that promote nerve regeneration also involve neurovascularization. Endothelial cells participate in angiogenesis and form a new intraneural vasculature in response to a hypoxic environment after injury, providing the migration surface for SCs. The regenerated vasculature also supplies nutrients and oxygen. Neurovascularization, the reconstruction of the microvascular network within a regenerated nerve, is a key link in nerve regeneration [155]. Mohammadi et al. [156] reported that a chitosan conduit with an uncultured stromal vascular fraction effectively improved peripheral nerve regeneration in rats with diabetes. Meiyuan Li et al. pre-vascularized skin precursor-derived Schwann cells (SKP-SCs)-loaded silk fibroin scaffolds subcutaneously and placed the pre-vascularized scaffolds inside the SKP-SCs loaded chitosan NGCs. The pre-vascularization strategy was demonstrated to be more beneficial for the revascularization of nerve regeneration than vascular endothelial growth factor (VEGF), achieving a nerve regeneration effect comparable to that of autologous transplantation [151] (Fig. 5D). Biologically, cell transplantation and in vivo/in vitro pre-vascularization are promising approaches for sprouting angiogenesis and vascular network remodeling, representing potential future directions for neovascularization [157].

#### 3.2.2 Chemical cues

**Fig. 6:**
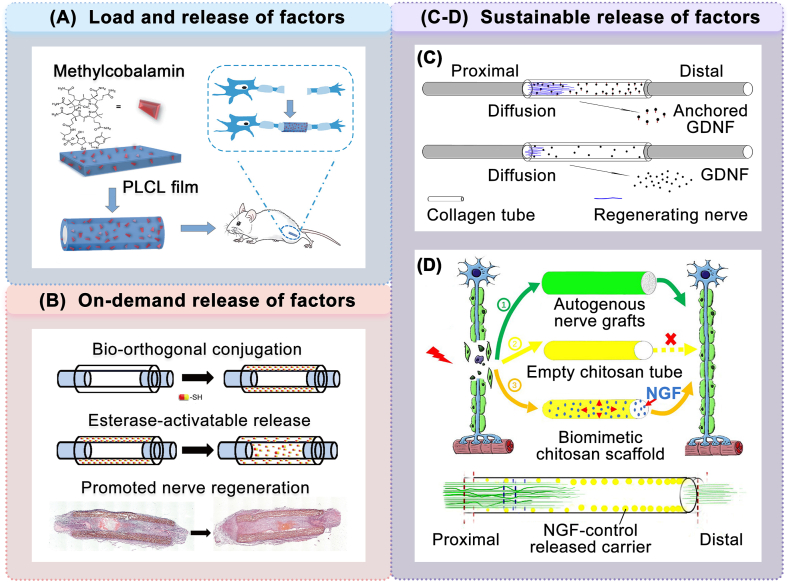
Chemical modification of NGCs prepared from FDA-approved materials in animal experiments. (A) Load and release of the chemical factors. Schematic of PLCL conduits loaded with different amounts of MeCbl (reproduced with permission) [158]. Copyright 2020, Wiley. (B) On-demand release of chemical factors. Schematic of a biologically orthogonal functionalized scaffold with esterase activation release (reproduced with permission) [162]. Copyright 2023, Elsevier. (C–D) Sustainable release of chemical factors. (C) Schematic of a collagen conduit for continuous delivery of GDNF (30 d) (reproduced with permission) [163]. Copyright 2018, Elsevier. (D) Schematic of a chitosan scaffold with long-term controlled release of NGF (8 weeks) (reproduced under the terms of the Creative Commons Attribution-NonCommercial-ShareAlike License (CC BY-NC-SA)) [164]. Copyright 2022, Wolters Kluwer.

Chemical cues involve the binding and delivery of biochemical factors or medications to the nerve repair scaffold. A number of studies have chemically modified NGCs prepared from FDA-approved materials. Zhang et al. developed a PLCL conduit that encapsulated and released different amounts of methylcobalamin, effectively restoring the injured sciatic nerve [158] (Fig. 6A). Sayanagi et al. fabricated a PGA conduit that encased electrospun nanofiber sheets loaded with mecobalamine (MeCbl), obtaining reparative efficacy comparable to that of nerve autografts [159]. Farahpour et al. enhanced sciatic nerve regeneration through the application of a chitosan conduit filled with acetyl L-carnitine solution [160]. Dong et al. developed a functionalized chitosan gel conduit loaded with deferoxamine (DFO), which accelerates nerve regeneration by promoting angiogenesis [161].

Researchers have achieved long-term and sustained delivery of chemical agents for up to two months. Ma et al. created a collagen scaffold that stably binds the glial cell-derived neurotrophic factor (GDNF) through chemical cross-linking. The scaffold provided a sustained delivery of factors exceeding 32 days, achieving a repair effect similar to that of nerve autografts [163] (Fig. 6C). Liu et al. developed a chitosan conduit capable of a continuous NGF release for eight weeks, leading to a restoring effect comparable to that of nerve autografts [164] (Fig. 6D). Moreover, researchers have explored the on-demand release of chemical factors from NGCs. In the study conducted by Wang et al., the thio-contained cysteine-tyrosine-isoleucine-glycine-serine-arginine was coupled to the chitosan conduits and released through esterase activation, which proved beneficial for nerve regeneration [162] (Fig. 6B). Additionally, the synergies of suitable chemical factors were evaluated and demonstrated to exceed the efficacy of a single chemical factor. Madduri et al. achieved the synergistic delivery of GDNF and NGF on collagen NGCs, surpassing the efficacy of single-factor applications [165]. Azizi et al. found that chitosan conduits incorporating both vitamin E and pyrroloquinoline quinone attained superior results compared to conduits with only one of the medications [166].

#### 3.2.3 Physical cues

Physical modification of the NGC is a key factor that contributes to nerve repair. Physical cues include topological morphology design [134] and the application of physical fields, such as electricity [167] and magnetism [32]. Topological cues have been extensively utilized. Hollow conduits that link the stump and facilitate nerve stem growth on a macro size may result in complications such as axon misalignment and inadequate nutrition permeability, prompting the development of 2D and 3D topological modifications for enhanced regulation of nerve regeneration on a micro-scale [168].

**Fig. 7:**
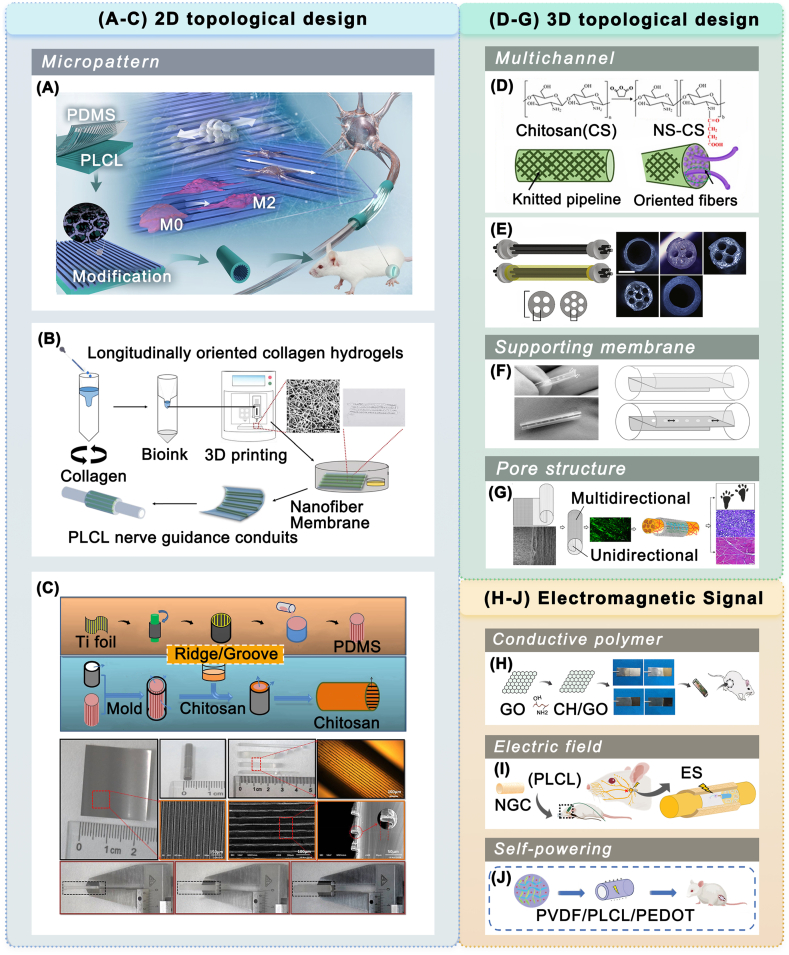
Physical modification of NGCs prepared from FDA-approved materials in animal experiments. (A–C) 2D topological cues. (A) Schematic of the PLCL conduit modified with ridge/groove micropatterns and surface-anchored GO nanosheet promoting nerve regeneration (reproduced with permission) [172]. Copyright 2020, American Chemical Society. (B) Fabrication flow chart of the PLCL conduit containing 3D printed longitudinally oriented collagen hydrogels on the inner surface (reproduced with permission) [170]. Copyright 2020, Royal Society of Chemistry. (C) Fabrication of the chitosan conduit with ridge/groove micropatterned inner wall and seamless side wall, with optical and scanning electron microscopy images of the micropatterned structures (reproduced with permission) [171]. Copyright 2018, Elsevier. (D–G) 3D topological cues. (D) Schematic of the multi-channel nerve conduit based on chitosan and chitosan derivatives (reproduced with permission) [173]. Copyright 2023, Elsevier. (E) Fabrication schematic and cross-sectional images of multi-channel collagen conduits with different numbers of channels (reproduced with permission) [171]. Copyright 2010, Elsevier. (F) Schematic of the chitosan conduit with longitudinal chitosan film introduced into the lumen (reproduced under the terms of the Creative Commons Attribution-NonCommercial-NoDerivatives 4.0 International License (CC BY-NC-ND 4.0)) [174]. Copyright 2016, Elsevier. (G) Schematic of the collagen conduit with unidirectional and multidirectional multilayered pore structure promoting nerve repair (reproduced with permission) [175]. Copyright 2021, Elsevier. (H–I) Electromagnetic signal cues. (H) Schematic of the chitosan/GO conduit fabricated by electrodeposition to repair a nerve defect and macroscopic diagram of the material surface morphology (reproduced under the terms of the Creative Commons Attribution-NonCommercial-ShareAlike License (CC BY-NC-SA)) [176]. Copyright 2023, Wolters Kluwer. (I) Schematic of PLCL conduits combined with direct current or charge-balanced pulse stimulation (pulse) to repair facial nerve injury (reproduced with permission) [177]. Copyright 2023, American Chemical Society. (J) Schematic of PVDF/PLCL/PEDOT self-powering conduits that facilitated the repair of injured peripheral nerves (reproduced with permission) [178]. Copyright 2024, Wiley.

The designs of 2D guide structures involve oriented surface micropatterns and aligned fibers [169]. Researchers have applied micropatterns of longitudinally oriented parallel ridge/groove structures based on collagen [170] (Fig. 7B), chitosan [171] (Fig. 7C), and PLCL [172] (Fig. 7A) materials to provide directional guidance for SC migration and axon extension. These micropatterned cues facilitate NGCs in achieving recovery results comparable to those of autologous transplantation.

3D topological designs refer to intraluminal and wall structures of conduits. 3D intraluminal structures can provide physical support and guidance, increase cell adhesion surface area, and prevent neural misalignment [179]. Studies have employed multi-channel [180,173] (Fig. 7D and E) and microchannel [181,182] structures based on collagen and chitosan scaffolds, achieving functional recovery similar to that of autologous transplantation. Porous support membranes have also been incorporated into chitosan conduits by Meyer et al. [174] (Fig. 7F) and Dietzmeyer et al. [183], supporting SC migration and promoting microvascular connectivity. These conduits achieved in vivo repair effects similar to those of autografts. In addition to small animal studies, researchers have tentatively implemented 3D intraluminal topological structures in large animal models and humans. Chitosan conduits filled with aligned PGA filaments were applied to treat long-distance nerve defects in Beagle dogs, obtaining restorative results comparable to those of autografts [184] Neuragen 3D, a collagen nerve conduit containing an orientationally aligned internal matrix, has been approved by the FDA [17], indicating further clinical application potential of topological modifications. Regarding 3D topological structure design, the wall structure primarily refers to the pore structure of the conduit wall. Cerri et al. [185] designed an NGC wall structure featuring pores with a radial gradient density. Radially oriented pores can restrict scar tissue formation and allow oxygen exchange. Millán et al. [175] developed a collagen scaffold with laminar flow-like pore structures. The inner layer with unidirectional pores promotes SC migration, while the outer layer with multidirectional pores inhibits the inward growth of fibroblasts. The modified conduits showed functional recovery similar to that of autografts (Fig. 7G).

**Fig. 8:**
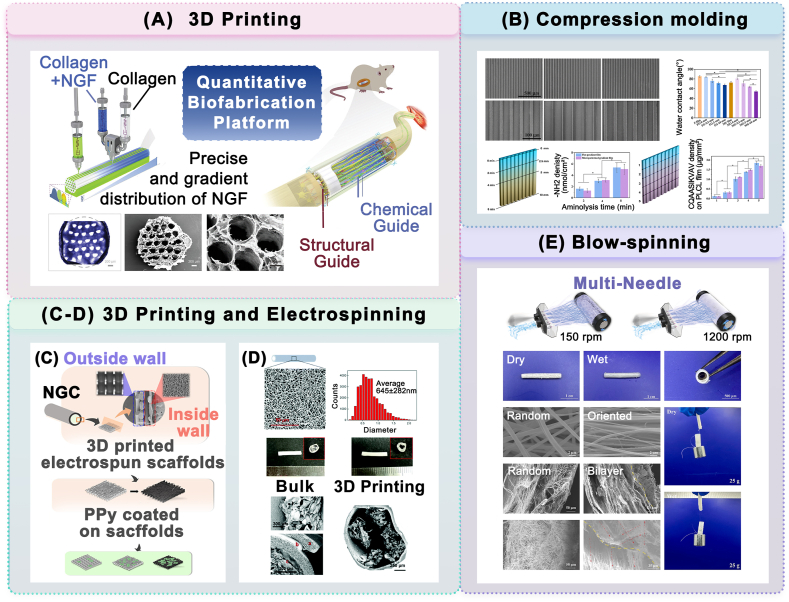
Biomanufacturing technology for physically modified structures of NGCs prepared from FDA-approved materials. (A) 3D printing technique. Schematic illustration and electron microscopy characterization of a collagen conduit with a high-precision microchannel structure and NGF gradient. (reproduced with permission) [67]. Copyright 2023, Wiley. (B) Compression molding technique. Fabrication routes and electron microscopy images of regular groove/ridged micropattern structures using a template thermo-pressing method (reproduced under the terms of the Creative Commons Attribution-NonCommercial-NoDerivatives 4.0 International License (CC BY-NC-ND 4.0)) [186]. Copyright 2022, Elsevier. (C–D) Electrospinning. (C) General images and electron microscopic images of suitable pore structures at nanometer/micron resolution in the inner wall of PLCL nerve conduit fabricated by electrospinning (reproduced with permission) [187]. Copyright 2022, American Chemical Society. (D) Schematic and electron microscopy of the nanopore structures with appropriate permeability of PLCL fiber scaffolds manufactured by electrospinning (reproduced with permission) [170]. Copyright 2020, Royal Society of Chemistry. (E) Blow-spinning. Schematic diagram and electron microscopic images of a double-layer collagen nanofiber conduit with internal fiber orientation and external fiber random arrangement prepared by multi-needle blow-spinning technique (reproduced under the terms of the Creative Commons Attribution-NonCommercial-NoDerivatives 4.0 International License (CC BY-NC-ND 4.0)) [188]. Copyright 2024, Elsevier.

The implementation of topological structures needs to be integrated with manufacturing technology. Fig. 8 delineates the fabrication techniques for various physical structures of the NGCs, encompassing traditional manufacturing technologies (freeze drying [171], solvent casting, compression molding [186], electrospinning [170,187], and blow-spinning [188]) and novel additive manufacturing technologies [67,170,187].

In addition to the extensive and prolonged exploration of topological cues, physical field-related strategies have gradually been enriched in recent years. During the late 20th and early 21st centuries, Schmidt et al. initially explored the effects of electrical stimulation on nerve cells and its possible mechanisms [189,190] Bioelectronic medicine has been extensively studied through the development of theories, materials, and devices. Electrical means have been reported to promote nerve growth and repair the peripheral nervous system [191,192]. Conductive polymers that can induce endogenous bioelectricity in the body have been utilized for nerve regeneration [38] Zhang et al. [172] anchored a PLCL scaffold surface with conductive graphene oxide (GO). The conduit achieved excellent regeneration with the integration of conductive polymer modifications and micron-scale grooves. Zhao et al. [176] prepared a chitosan/GO composite conduit. The incorporation of a conductive polymer enabled the conduit to attain a reparative effect comparable to that of autologous transplantation. Numerous studies have explored the impact of external electromagnetic fields on peripheral nerve regeneration (Fig. 7H). Mohammadi et al. [156] combined chitosan conduits with pulsed electromagnetic fields (PEMF). The short-term systematic exposure to PEMF promotes nerve reconstruction, possibly through its effects on neurotrophic factor levels. Song et al. [193] applied direct current electrical stimulation (ES) to a polypyrrole (PPY)/PLCL conduit. The approach demonstrated comparable efficacy to the autologous transplant group, probably owing to the positive influence of the electric field on SC migration and neurite outgrowth. Choe et al. [177] employed transcutaneous electrical nerve stimulation with PLCL conduits to repair damaged facial nerves in rats, finding that direct and pulsed currents enhanced nerve regeneration without causing side effects (Fig. 7I). Further, NGCs capable of self-producing electricity have been developed in the latest research. Wang et al. [178] incorporated conductive polymer poly(3,4-ethylene dioxythiophene) (PEDOT) and inductive piezoelectric polymer polyvinylidene difluoride (PVDF) into PLCL to fabricate composite nerve guidance conduits (NGCs) exhibiting both spontaneous and conductive properties (Fig. 7J). Conduits can attain a nerve repair outcome equal to autologous nerve transplantation through the modulation of the immune microenvironment. In addition, self-powered technology combined with microneedle technology has been innovatively applied in nerve regeneration. Hu et al. [194] combined piezoelectric, microneedle, and topological guidance techniques to develop a self-powered NGC for sea cucumber bionics. The microneedle tips on the outer layer can generate microcurrents and enhance fixation; the microchannel structure in the inner layer can guide the axon extension. Zhang et al. [195] developed the self-powered NGC by integrating enzymatic biofuel cells and microneedles. The microneedles inside the conduit can be loaded with different enzymes to generate physiological microcurrents and enhance self-repair capabilities. These newly created technologies warrant further exploration and are anticipated to be utilized in conjunction with more approved or optimal materials.

In summary, the predominant modification methods at the physical level involve characteristic structures based on morphological cues. Topologies, especially micro-nano topology structures, modulate cell and protein behavior at the injury site by mimicking the physical microenvironment of natural tissues, thus facilitating nerve regeneration. Systematic and validated topological designs based on suitable materials can achieve satisfactory stability of scaffold morphological structures in vitro and in vivo [169,196] The incorporation of electromagnetic stimulation has lately unveiled further possibilities. However, it still necessitates stringent and exact regulation to address the challenges posed by invasive procedures and electromagnetic radiation [23,197,198].

#### 3.2.4 Combined cues

To promote nerve regeneration after injury, numerous studies have adopted flexible combinations of biological, physical, and chemical cues on NGCs prepared from FDA-approved materials.

**Fig. 9:**
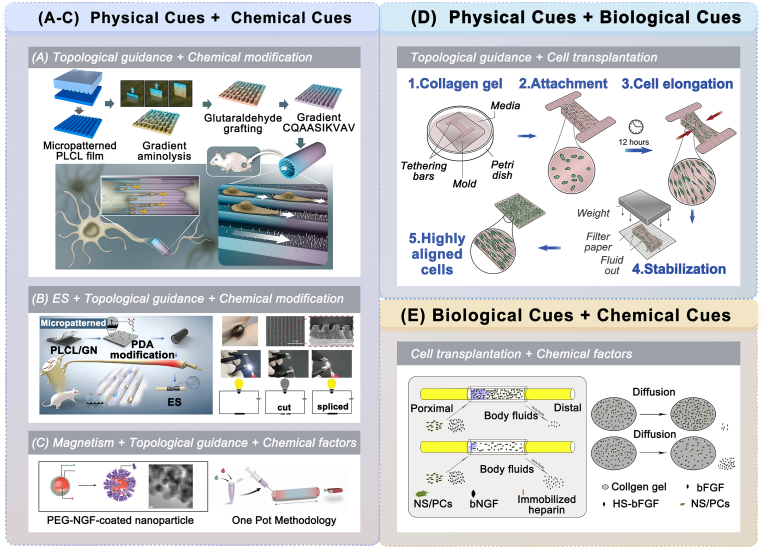
Combined modification of NGCs prepared from FDA-approved materials in animal experiments. (A–C) Combination of physical and chemical cues on NGCs. (A) Schematic of the fabrication of ridge/groove micropatterned PLCL conduit with gradient-density CQAASIKVAV peptide modification (reproduced under the terms of the Creative Commons Attribution-NonCommercial-NoDerivatives 4.0 International License (CC BY-NC-ND 4.0)) [186]. Copyright 2022, Elsevier. (B) Schematic representation and conductivity properties of the PLCL/graphene scaffold incorporating ridge/grooved micropatterns, polydopamine modification, and ES (reproduced with permission) [201]. Copyright 2023, Wiley. (C) Schematic of magnetic nanoparticles modified with NGF (MNP-NGF) and collagen conduits (Neuragen) containing magnetically aligned gels with anisotropic MNP-NGF distribution (reproduced with permission) [202]. Copyright 2021, Wiley. (D) Combination of physical and biological cues in NGCs. The fabrication flow diagram of a 3D collagen hydrogel scaffold containing highly aligned SCs (reproduced Creative Commons Attribution 3.0 Unported (CC BY 3.0)) [203]. Copyright 2013, Elsevier. (E) Combination of biological and chemical cues in NGCs. The schematic of the collagen conduit with anchored bFGF and transplanted neural stem/progenitor cells (NS/PCs) (reproduced with permission) [199]. Copyright 2017, Elsevier.

Several studies have investigated the biological and chemical modifications of NGCs. Ma et al. conducted a nerve guidance conduit based on rat tail collagen that introduces neural stem cells and promotes the proliferation and survival of neural stem cells by binding basic fibroblast growth factor (bFGF) to the conduit with heparin cross-linking. The experimental groups achieved functional and morphological results similar to autologous transplantation by combining conduits with cells and cytokines [199] (Fig. 9E). Zhang et al. implanted mesenchymal stem cells (MSCs) via erythropoietin-loaded chitosan nerve conduit, promoting defective nerve healing [200].

Regarding the fusion of physical and biological cues, numerous studies designed specific 3D topographic structures of the matrix to realize the interaction of physical forces between cells and the 3D matrix, thus constructing biological scaffolds with aligned seed cells and promoting nerve regeneration in different cases. Georgiou et al. performed a 3D culture of SCs in a tethered type-1 collagen hydrogel to induce the anisotropic structure of the biological scaffold via cell-matrix interactions. The tension generated by the cells caused a uniaxial strain in the gel, forming a composite scaffold containing oriented aligned SC columns. These structures were rolled into a rod-like form and filled in the collagen conduits, leading to repair results comparable to those of autografts [203]. (Fig. 9d) Next, the researcher attempted to employ SC-like differentiated adipose-derived stem cells (dADSCs) to construct similar biological scaffolds due to the limited availability of SCs. The mimicking effect of aligned dADSCs on Büngne bands was conducive to directed neuronal growth and elongation [204] Additionally, Rayner et al. developed human stem cells, a differentiated clinical-grade clonal human stem cell line (CTX0E03), to build 3D scaffolds similar to those described above. The scaffold was encased in a collagen membrane on the outside and a tethered collagen hydrogel containing aligned cells inside. This resulted in a nerve regeneration effect similar to that of an autograft in athymic nude mice [205]. In addition, the topology technology based on microspheres is also combined with seed cell transplantation. Lately, Zhu et al. [206] filled the chitosan NGC with 3D chitosan microspheres characterized by a textured surface with a uniform distribution of micropores to load ADSCs. The topological structure provided increased surfaces for the adhesion and proliferation of exogenous ADSCs, thereby promoting a repair effect similar to that of autografts.

For the combination of physical and chemical modifications, most studies have employed the combined application of topological structures and chemical factors. Cao et al. [207] and Cui et al. [208] developed linearly ordered collagen scaffolds with oriented filaments bound to neurotrophic factors, promoting nerve repair in rat and pig models. Wang et al. [67] used 3D printing to fabricate a collagen conduit featuring a high-precision microchannel structure and longitudinal gradient distribution of NGF. This bioprinted conduit exhibited excellent repair performance both in vitro and in vivo. Yu et al. [209] and Zhang et al. [186] (Fig. 9A) concluded that the combination of peptides and ridge/groove surface micropatterns within the PLCL conduit significantly promotes nerve regeneration. Similar applications have been demonstrated in nerve repair using chitosan conduits. Rao et al. developed chitin conduits using oriented chitosan nanofiber hydrogel grafted with an RGI/KLT peptide mixture (ACG-RGI/KLT), achieving outcomes comparable to those of autografts [210]. Qi et al. used a mixture of chitosan and the extracellular matrix derived from human bone mesenchymal stem cells (BMSC-dECM) to fabricate the internal directional fibers within the external acetylated chitosan scaffold, providing nutritional and directional guidance for axon regeneration [83]. Zhang et al. prepared chitosan conduits containing aligned chitosan fibers. The aligned fibers were modified with ZIF-8 nanoparticles that can release Zn2+. This conduit regulated the inflammatory microenvironment and guided axon extension, achieving a repair level comparable to that of autologous transplantation [211]. Furthermore, studies have combined electromagnetic and chemical cues to modify NGCs. Antman-Passig et al. loaded a collagen gel containing NGF-conjugated magnetic nanoparticles into a type-I collagen conduit. The control of a remote magnetic field promoted the anisotropic arrangement of magnetic nanoparticles, which was conducive to the directional development of axons [202] (Fig. 9C). Deng et al. developed a chitosan conduit containing carboxymethyl chitosan/polyaniline conductive hydrogels loaded with 7, 8-dihydroxyflavonoids, attaining repair effects comparable to those of autografts [212]. Lu et al. designed a polydopamine-modified PLCL/graphene scaffold with ridge/grooved surface micropatterns and applied it in combination with ES. The integration of multiple topology, electricity, and chemistry cues significantly assisted nerve repair in vivo [201] (Fig. 9B).

Overall, scaffolds prepared from FDA-approved materials have been combined with various modifications to develop novel NGCs in animal studies (Table 2). Different strategies have their strengths and limitations. For biological strategies, exogenous seed cell transplantation provides fresh and powerful regenerative forces at the repair site. It can replenish deficient repair cells to regulate the local microenvironment and directly participate in the repair processes [213]. However, seed cell transplantation presents many problems, such as tedious identification, low post-transplant survival, immune rejection, tumorigenesis, and ethical regulations [214,215]. Chemical strategies involve the application of biochemical factors individually, in combination, continuously, or sequentially. Biochemical factors promote cell proliferation and differentiation, as well as assist in the regulation of inflammation and immunity. Compound or multiple time-series releases of biochemical factors can facilitate repair in a biomimetic manner by simulating the temporal course of the factor expression. However, bioactive factors, including proteins and peptides, are susceptible to the interference and destruction of external conditions. They require stringent environmental conditions (such as low temperatures) to maintain their activity during production, storage, and transportation [216]. In clinical applications, maintaining a stable activity and safe dose of cytokines while achieving accurate release is challenging. As for physical cues, multidimensional topology modification can help achieve specific tissue-guiding functions by regulating cell behavior and fate at cellular and molecular levels [217,218]. Topologically engineered and modified conduits do not impose stringent requirements in terms of preservation, transportation, and application, unlike biologically or chemically modified conduits. Moreover, they are not required to confront the ethical risks associated with cell transplantation and delivery of biologically active factors [219]. Biological or chemically modified conduits are considered combination products by the FDA from a regulatory perspective. These products can be integrated into biological products, drugs, and other devices. Therefore, they are subject to more complex and stringent regulatory requirements than medical devices [220]. The independent application of physical topological modifications of devices avoids the additional time and costs associated with the testing and review of medications and biologics. As a result, physically modified NGCs are more likely to complete a controlled in situ nerve repair process and are suitable for the near-term development of clinical translation. Multi-scale topologies are promising for human applications, while the clinical applications of wireless electromagnetic radiation require further investigation.

**Table 2:** Applications of FDA-approved materials coupled with multiple modifications.

Strategies	Content	Advantages and Roles
Biological modification	Inoculation of SCs and stem cells of different origin	Act as a source of deficient cells during repair, regulate the microenvironment, and pre-vascularize and simulate the formulation of bands of Büngner
Chemical modification	Long-term stable and controlled local release of factors and medications	Act as an activator to promote angiogenesis, SC migration and proliferation, and neuronal differentiation
Physical modification	3D structures and 2D micropatterns to guide orientation: longitudinally oriented multi-channels, microchannels, parallel fibers and parallel ridge/groove structures	Mimic natural nerve tissue, provide a good growth path and microenvironment support for axon, SC and EC, promote directional nerve growth, and prevent diffuse growth
	3D structures with guiding and material exchange properties: intracavitary longitudinal porous film guidance structure	Provide physical support for SC migration and promote two-compartment microvascular communication and nutrient exchange
	Porous structure: internal and external anisotropic pore structures with micrometer to nanometer scale morphology and controllable aperture gradients and orientations	Promote proliferation and migration of regenerative cells, promote nutrient exchange, and prevent the formation of fibrous scar tissue
	Corrugated outer wall structure	Improve flexibility/bendability for nerve reconstruction in highly mobile areas with frequent squeezing and twisting, such as muscles and joints
	Physical signals: combined with ES, magnetic stimulation or PEMF	Affect the level of biochemical factors in nerve tissue and promote the growth of nerve cells and the directional growth of axons

∗ Abbreviation: Schwann cell (SC), Endothelial cell (EC).

## 4 Outlook

**Fig. 10:**
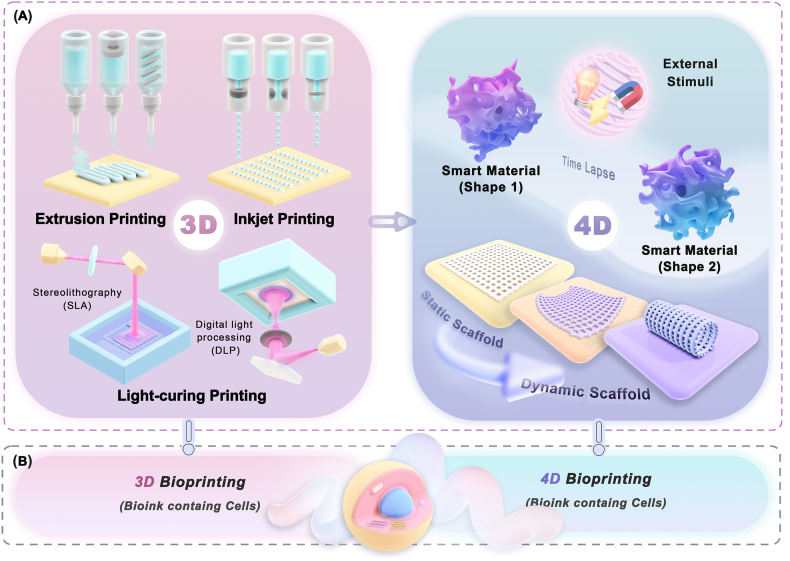
Schematic representation of future advanced additive manufacturing technologies adapted for commercial development of NGC. (A) Inanimate 3D/4D fabrication techniques are suitable for the construction of NGCs modified with physical strategies in the short term. (B) 3D/4D biofabrication techniques are suitable for the construction of multifunctional NGCs modified with combined strategies in the long term.

Based on the above in-depth discussion of biomaterials and additional strategies, a future development path for the commercialization of NGC is envisioned. In the process of promoting product commercialization, "material optimization" is a basic and consistent means, while "application of additional modification strategies" is the key phased task in dynamic adjustment. In the short term, physical topological modification should be the dominant option in modification strategies for commercializing NGCs. In the long run, the more complete and comprehensive composite modification strategy will be applied after the actual risks and barriers to clinical application have been broken down. Under phased targets, the implementation of additional strategies is inseparable from the involvement of advanced manufacturing technologies as necessary tools (Fig. 10).

### 4.1 The direction for short-term clinical translation

Physical modifications of NGCs should be investigated in the preliminary stages. In a clinical scenario, the site, size, nature, and complexity of a nerve injury vary from patient to patient, as does the specific anatomy of the damaged nerve and the tissue morphology within the nerve bundle. Therefore, given the complexity and precision of the nervous system, ideal nerve repair requires personalized and precise bionic treatments [221]. Simulated reconstruction and manufacturing reproduction of 3D anatomical structures are key approaches for customized and accurate restoration.

#### 4.1.1 Simulated reconstruction

Imaging techniques are crucial for the simulated reconstruction of damaged neural structures. Clinical imaging techniques including ultrasound scan (US) and magnetic resonance imaging scan (MRI) can provide an initial morphological assessment of the injured nerve [222]. On this basis, 3D imaging technology such as 3D structured light scanning (SLS) can reconstruct the morphological structure of complex geometric objects in 3D [39]. The combination of various imaging techniques helps to display and simulate the size and shape of natural nerves, setting the stage for custom-printed scaffolds that match the damaged nerve.

#### 4.1.2 Manufacturing reproduction

In terms of manufacturing reproducibility, traditional molding techniques are limited in their ability to accurately fabricate complex microscale topological structures. Therefore, the application of advanced additive manufacturing techniques is of great importance in implementing multi-scale terrain guidance and simulating fine neural tissue structures. The following is a discussion of additive manufacturing techniques applied in nerve regeneration, focusing on techniques that have used FDA-approved and predicted desirable materials as printing materials (Fig. 10A).

##### 4.1.2.1 Extrusion-based 3D printing

Extrusion-based printing (e.g., fused deposition modeling (FDM), direct ink writing (DIW)) was the earliest additive manufacturing technique used in the fabrication of NGCs [223]. This method involves extruding viscous ink through a nozzle and depositing it on a substrate [224] making it simple, inexpensive, and highly scalable [225]. Several studies have applied FDA-approved conduit materials to the extrusion process, achieving the fabrication of NGCs and their micro-scale guide structural sections. Wang et al. constructed a mechanical analytical model of collagen and fabricated collagen conduits with intrinsic longitudinal high-density microchannel arrays to mimic natural neural structures [67]. Yoo et al. used acidified type I collagen as a bio-ink and formed the longitudinal-oriented collagen hydrogel guide structure on a PLCL electrospun membrane scaffold [170]. Studies have been conducted on the preparation of NGC using extrusion-based printing with potentially ideal biomaterials. Yao et al. used FDM technology to print micrometer-scale PCL filaments on rotating rods to construct PCL scaffolds with appropriate mechanical properties [226]. Using PCL as one of the printing materials, Johnson et al. customized the NGC to match the complex shape of the bifurcated neural pathway using a micro-extruder-based 3D printing system [39]. However, due to the complex rheological behaviors of extruded materials [227], it faces challenges in achieving higher printing accuracy and resolution [228].

##### 4.1.2.2 Inkjet-based 3D printing

Inkjet-based printing uses piezoelectric or thermal actuators to spray bio-ink onto a substrate from a nozzle in the form of tiny droplets of picoliter and deposit them at a pre-set location [229,230]. This technique is a contact-free, mask-free, low-cost, material-saving numerical control technique with higher printing speed and resolution than the extrusion process [231,232]. Early studies have been tentatively conducted using FDA-approved conduit materials for inkjet-based printing [233]. However, the technique has high requirements on the properties of the ink flow [234], and sacrifices higher printing accuracy to reduce nozzle clogging [230]. Electrohydrodynamic (EHD) jet printing is an emerging material injection technology that uses a strong electric field over a short distance to drive the ink to produce the liquid cone (Taylor cone) followed by the very fine liquid jet [235]. The EHD jet printing system can eject tiny droplets of submicron size and precisely deposit micron/submicron scale fibers. The melt-based EHD jetting process is also called melt electrowriting (MEW) [236], which enables more microscopic and accurate tuning of fiber size and positioning [237]. This technique is used on NGCs made of potentially ideal biological materials, enabling the fabrication of ultra-fine fibers with high orientation and controllability. Li et al., Zhang et al. and Fang et al. used EHD/MEW technology to fabricate PCL-based patterned scaffolds characterized by ultra-fine fibers with a minimum diameter of ∼10 μm [6,217,238]. EHD jet printing, as a relatively novel technique capable of customizing high-resolution controllable fiber structures, has been increasingly applied to neural tissue engineering.

##### 4.1.2.3 Light-curing 3D printing

Light-curing 3D printing involves selectively curing photosensitive polymers with UV light, providing faster printing speed and higher printing resolution compared to traditional extrusion printing and inkjet printing [239]. Based on the different ways of light scanning, the printing technology is mainly divided into stereolithography (SLA) and digital light processing (DLP) [240]. SLA scanning point by point [241] can fabricate micro-grooved (10–30 μm) structures of NGCs to provide topography guidance [242]. DLP curing the entire single layer attains higher printing efficiency and fidelity (∼80 %) [243] in nerve repair, with resolutions up to 1–50 μm [241,244].

However, this technology is limited to light-sensitive materials. Modification by introducing photocrosslinking groups such as methacrylic anhydride (MA) is a common way of conferring photocrosslinking properties on polymers [245]. Currently, the common bioink used for fabricating NGCs with DLP are gelatin methacryloyl (GelMA)-based complex polymer [246,247]. The ideal polymers modified with MA, including methylacryloylated PCL (PCLMA) and methylacryloylated silk fibroin (SilMA), have been demonstrated to have sufficient mechanical properties and show potential for in-vivo repair [248,249]. Singh et al. printed multi-channel NGCs (diameter: 500 μm) from photocrosslinkable PCL by SLA technique and achieved excellent regeneration [248]. Wu et al. fabricated SilMA/GelMA conduits with excellent mechanical properties and cell adhesion properties using DLP technology [250]. Light-curing 3D printing can further optimize the mechanical properties and stability of printed products with more desirable printing materials, which can help to produce more sophisticated and intelligent NGC.

##### 4.1.2.4 4D printing

Adding time as a new dimension on the basis of 3D manufacturing gave birth to 4D printing technology. 4D printing refers to using smart materials to create dynamic structures that respond to external stimuli. Researchers can program materials to drive procedural and parametric deformations of printed products. In the field of nerve regeneration, several studies have used 4D techniques to self-curl the material to form a conduit structure in vivo, which is beneficial for simplifying surgical procedures and wrapping implanted conduits around nerve stumps [251,252]. Furthermore, given that nerve regeneration is a complex process involving multiple events, the application prospects of 4D techniques lie in regulating nerve repair in multiple stages. Sun et al. designed a responsive directional surface micro-groove morphology to sequentially regulate the neural regeneration process [253]. Looking ahead, 4D techniques can be used to program and dynamically regulate topological micromorphologies based on smart materials, thus participating in and facilitating different stages of nerve restoration.

In the laboratory phase, guided by the strategies of simulated reconstruction and manufacturing reproduction, customized platforms have been created for NGCs with personalized physical structures. In the clinical phase, a more comprehensive production chain needs to be established. The systematic and mature industrial production of the commercial conduit requires the construction of networking, digitalization, and intelligence. It is necessary to integrate optical imaging, digital modeling, computer-aided design, and additive manufacturing technology to realize precision machining. Further expansion and application of ultrasonic printing [254], five-dimensional (5D) printing, and six-dimensional (6D) printing [255], in addition to the already-applied technologies, is anticipated to accomplish a deep integration of manufacturing technology with biological systems in the future. By the enhancement of cost control and quality supervision based on this novel production mode, large-scale and customized production of physically modified products is made possible. In all, rapid, accurate, and programmable nerve repair is expected to be achieved through the optimization of conduit materials and the improvement of physical modification strategies. This is a crucial development direction for the commercialization of NGCs in the short term.

### 4.2 The direction for long-term clinical translation

Based on animal studies to date, the application of composite add-on strategies has shown a growing trend. The combination and interplay of biological, chemical, and physical factors results in a better repair effect. Advanced 3D bioprinting of assembled cells [99] and 4D bioprinting of dynamically imposed regulation [256] have been used to refine the composite additional strategies for promoting nerve regeneration. However, NGCs with combined additional modification strategies cannot be rapidly translated into clinical medical devices in a short period of time. Despite their widespread use in animal research, seed cells and biochemical factors may still face great obstacles and challenges in short-term clinical applications due to the limitations of scientific techniques and ethical risks. In the long-term future, the dilemmas and barriers to the clinical application of combined strategies are expected to be overcome following advances in regenerative medicine technology and improvements in regulatory systems. At that time, regenerative scaffolds modified with combined strategies will find stable applications in clinical practice. After all, it is tough to construct a bionic scaffold that mimics complex neural tissue using a single manufacturing process and a single material. With the progress of 3D/4D fabrication techniques that integrate multi-process, multi-material, multi-scale, multi-factor, multi-cell, and multi-time series, the construction of multifunctional biological scaffolds modified by composite strategies is an inevitable development trend in the long term (Fig. 10B).

In summary, targeted studies based on core elements are required to obtain commercially available NGCs with improved efficacy in clinical practice. The optimization and development of materials is a constant fundamental element. The phased application of appropriate modification strategies based on FDA-approved or, more ideally, optimized materials is another key element. Previous studies and the above analysis have reached the conclusion as follows. In the short term, physical modification through innovative imaging and additive manufacturing techniques may be a fast path to realizing new clinical applications. In the long run, with the rapid development of tissue engineering technology and the continuous improvement of medical ethics, the compound superposition of multiple modification strategies will be an inevitable trend.

**Fig. 11:**
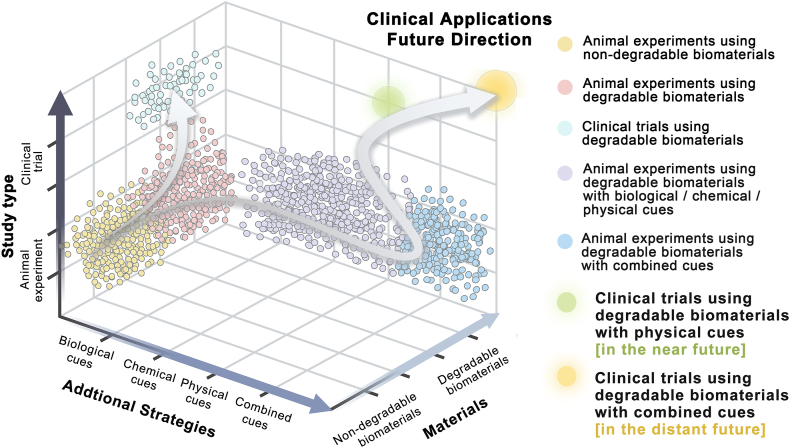
The historical process and development trend of nerve guidance conduits. Potential strategies to achieve future clinical translation are highlighted with glowing dots.

Looking at the past and focusing on the present can help to better envision the future. Clinical evaluation has comprehensively reviewed the repair efficacy of hollow NGC which developed over a long period of time. The bibliometric analysis of current laboratory research has revealed the key factors affecting the outcome of nerve regeneration. Through theoretical analysis of design policies and in-depth discussion of manufacturing techniques, important insights can be generated on the fundamental paths and important directions for future commercialization. The historical development of NGC's clinical translation and possible development paths for its future commercialization are summarized in Fig. 11. We hope to provide a macro perspective and decision-making basis for researchers who are concerned about commercialization, as well as help them better grasp the research and development goals and directions. To promote better patient recovery, we advocate for strategic, incremental clinical application of improved commercial products based on existing FDA-approved products. Thus, this review has made an outstanding contribution to guide the development of NGC research and shed light on the direction of NGC commercialization.

## 5 Conclusion

This review interprets the dynamic process of NGC commercialization from the perspective of longitudinal development from laboratory to clinical. The analysis of FDA-approved NGCs suggests that they may have a good efficacy comparable to autograft. Six months after surgery may be a critical window period for clinical observation. In addition, the progress of laboratory NGCs under development is reviewed, with an in-depth discussion of two key elements affecting nerve regeneration: biomaterials and additional modification strategies. Based on the analysis of current clinical and laboratory manifestations of NGCs, this study provides an outlook on future development pathways and manufacturing strategies for the commercialization of NGCs. For the development path, material optimization is the constant and fundamental means. Based on FDA-approved or, superior materials, the progressive implementation of add-on strategies is the key task, with early physical cues as a priority and long-term combined cues as leadership. Rapid progress toward commercialization requires dynamic integration with more advanced manufacturing technologies, including 3D/4D printing. Therefore, this review has made an outstanding contribution to guide the development of NGC research and shed light on the direction of NGC commercialization.

## CRediT authorship contribution statement

**Chundi Liu:** Writing – review & editing, Writing – original draft, Visualization, Supervision, Resources, Investigation. **Mouyuan Sun:** Writing – review & editing, Supervision, Software, Methodology, Funding acquisition, Formal analysis. **Lining Lin:** Project administration, Investigation, Data curation. **Yaxian Luo:** Validation, Resources. **Lianjie Peng:** Methodology, Data curation. **Jingyu Zhang:** Methodology, Investigation. **Tao Qiu:** Visualization, Validation. **Zhichao Liu:** Resources, Formal analysis. **Jun Yin:** Writing – review & editing, Supervision, Funding acquisition, Formal analysis. **Mengfei Yu:** Writing – review & editing, Supervision, Software, Project administration, Funding acquisition, Conceptualization.

## Declaration of competing interest

The authors declare that they have no known competing financial interest or personal relationships that could have appeared to influence the work reported in this manuscript.

## Data Availability

Data will be made available on request.
