# Supplementary material for: Potentially commercializable nerve guidance conduits for peripheral nerve injury: Past, present, and future
Source: Mater Today Bio. 2025 Feb 5;31:101503. doi: 10.1016/j.mtbio.2025.101503 (PMC11867546; doi:10.1016/j.mtbio.2025.101503)
Supplement: Multimedia component 1 [file mmc1.pdf]

Supporting Information

Literature search and selection

Formula-based literature searches were conducted in several electronic databases, including PubMed (MEDLINE), Embase, Web of Science, and Cochrane Central Register of Controlled Trials (CENTRAL). All English-language publications from January 1, 2000, to June 1, 2024, were searched for articles on clinical trial types that were not restricted by country. The literature-screening process is illustrated in Fig. S1A. The following search terms were used:

PubMed: ("conduit*" OR "nerve guide" OR "nerve guidance" OR "nerve guide conduit*" OR "nerve tube") AND ("Peripheral Nerve Injuries"[MeSH Terms] OR "nerve defect*" OR "nerve repair" OR "nerve regeneration" OR "nerve reconstruction" OR "nerve gap" OR "nerve surgery") AND ("digital" OR "hand")

Embase: ('nerve guidance conduit'/exp OR 'nerve guidance conduit*' OR 'conduit') AND ('peripheral nerve injury'/exp OR 'peripheral nerve injur*' OR 'nerve surgery'/exp OR 'nerve surgery' OR 'nerve regeneration'/exp OR 'nerve regeneration' OR 'nerve reconstruction'/exp OR 'nerve reconstruction') AND ('digital nerve'/exp OR 'digital nerve injury'/exp OR 'digital')

Web of Science:

#1: (((((TS=("nerve guidance conduit*")) OR TS=("nerve guide")) OR TS=("nerve guidance")) OR TS=("nerve guide conduit*")) OR TS=("nerve tube")) OR TS=("conduit")

#2: ((((((TS=("peripheral nerve injur*")) OR TS=("nerve gap")) OR TS=("nerve defect*")) OR TS=("nerve surgery")) OR TS=("nerve regeneration")) OR TS=("nerve repair")) OR TS=("nerve reconstruction")

#3: (TS=("digital")) OR TS=("hand")

#1 AND #2 AND #3

Cochrane Central Register of Controlled Trials:

#1: MeSH descriptor: [Peripheral Nerve Injuries] explode all trees

#2: MeSH descriptor: [Nerve Regeneration] explode all trees

#3: "nerve guidance conduit*":ti,ab,kw OR "nerve guide":ti,ab,kw OR "nerve guidance":ti,ab,kw OR "nerve guide conduit*":ti,ab,kw OR "nerve tube" OR "conduit*":ti,ab,kw) AND ("nerve repair":ti,ab,kw OR "nerve reconstruction":ti,ab,kw OR "nerve defect*":ti,ab,kw OR "nerve gap":ti,ab,kw OR "nerve surgery":ti,ab,kw

#4: "digital":ti,ab,kw OR "hand":ti,ab,kw

#5: {OR #1-#2}

#3 AND #4 AND #5

During the selection process, two reviewers independently assessed the eligibility of the studies in a nonblinded, standardized manner. Differences between the reviewers were resolved by reaching a consensus.

Quality assessment

Two reviewers performed independent and duplicate risk-of-bias assessments for the included studies. For different types of clinical trials, the reviewers jointly decided to use the Cochrane risk-of-bias tool 2 (RoB 2) for randomized controlled trials, Newcastle-Ottawa Scale (NOS) for cohort studies, and Joanna Briggs Institute (JBI) critical appraisal tools for case series. The quality assessment results were charted. The results of the RoB 2 tool for five randomized controlled trials, NOS for two cohort studies, and JBI critical appraisal tools for six case series have been graphed and displayed in Fig. S1B. All the included studies were assessed as not high-risk, and most were low-risk studies.

Data extraction

Two authors independently extracted the data from the included studies. Disagreements were resolved by requesting another reviewer to review the research information and by discussions among the multiple reviewers. The following data were extracted: authors, year of publication, country in which the study was conducted, study duration, inclusion and exclusion criteria, total number of participants included in the study, total number of injured

nerves included in the study, mean age of the study participants, site of nerve injury, mean length of nerve injury gaps, interventions, controls, duration of follow-up, and outcome indicators. These data are summarized in Fig. 2A.

Data synthesis and Statistical analysis

Quantitative and qualitative data were collated separately in Excel 2007 and pooled into stata16 for meta-analysis. For continuous variables, the effect size was expressed as mean with 95% confidence intervals (*CIs*) and assessed using a fixed-effects model. For categorical variables, the effect size was expressed as a ratio with a 95% *CI* and evaluated using a random-effects model. The results are presented as forest plots. Heterogeneity among the study outcomes was assessed using the I^2 index and Cochran's Q statistics.

Outcome Index and Evaluation Criteria

The s2PD is a static tactile examination method in which a static two-point stimulus is applied to the participant's skin, and the shortest distance between the two points identified by the participant is measured. According to the criteria of the Nerve Injuries Committee of the British Medical Research Council modified by Mackinnon and Dellon, s2PD was classified into different grades: S4 (≤6 mm), S3+ (7–15 mm), S3 (>15 mm) and worse than S3 (cannot distinguish between two static points). Based on these parameters, s2PD recovery was classified into three levels: excellent (S3+ and S4), good (S2+ and S3), and poor (worse than S3). Meaningful s2PD recovery was defined as S4 and S3+, that is, equivalent to an s2PD of no more than 15 mm.

The m2PD is a method of tactile measurement in which a moving two-point stimulus is applied to the participant's skin and the shortest distance between the two points perceived by the participant is measured. m2PD was rated ‘excellent’ if it was no more than 4 mm and as ‘good’ if it was 5–7 mm. Meaningful recovery of m2PD was defined as m2PD not exceeding 7 mm.

Semmes–Weinstein monofilament (SWMF) test is a neurological test to detect sensory loss in the skin, in which monofilaments of different specifications (including number and force) are applied to the participant’s skin, and the lightest monofilament specifications allowing participants to produce a normal tactile response are scored and classified according to the following criteria: normal (fiber number [1.65–2.83], force [0.008–0.07 g]); diminished light touch (DLT) (fiber number [3.22-3.61], force [0.16–0.4 g]); diminished protective sensation (DPS) (fiber number [3.84–4.31], force [0.6–2 g]); loss of protective sensation (fiber number [4.56–6.45], force [4–180g]); loss of deep pressure sensation (fiber number [6.65], force [300 g]); untestable (no response) (fiber number [>6.65]). Meaningful recovery of SWMF was defined as normal, and the DLT was equivalent to a threshold of less than 0.4 g.

Bibliometrics analysis

The literature source was the core collection of the Web of Science. The search strategy was as follows: TS = [“peripheral nerve injury” AND “conduit”]. The search time was limited from January 1, 2000, to June 1, 2024, excluding patents, conference abstracts, and book chapters. After excluding duplicate articles, 1392 references were included. A comprehensive visual analysis was conducted using keywords from the literature.

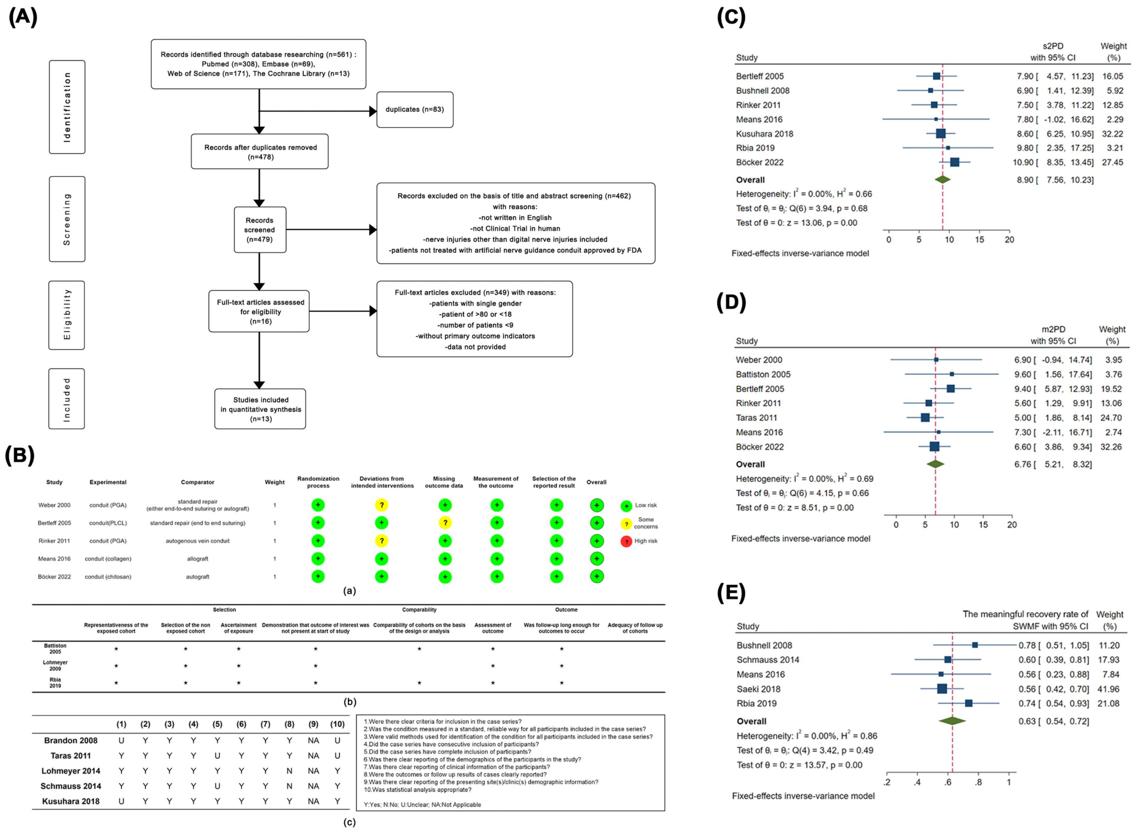

Figure S1. (A) Flow diagram of literature search and selection. Thirteen trials were included for analysis. (B) The reviewer's judgment of each risk of bias item in all included studies. (C-E) Forest plot. The effect of FDA-approved nerve guidance conduits on s2PD (C), m2PD (d), and SWMF (E). FDA, U.S. Food and Drug Administration; SWMF, Semmes–Weinstein monofilament; CI, confidence interval.
